# An Unusual and Discrepant Presentation of a Skull Base Paraganglioma

**DOI:** 10.7759/cureus.66394

**Published:** 2024-08-07

**Authors:** Raj Patel, Yusuf A Allam, Mohammad K Shukairy, Matthew Kircher

**Affiliations:** 1 Otolaryngology, Loyola University Medical Center, Maywood, USA; 2 Anatomical Sciences, University of Michigan, Ann Arbor, USA

**Keywords:** clinical neurotology, neuroendocrine tumors (nets), head and neck cancer surgery, neurogenic tumor of head and neck, isolated hypoglossal nerve palsy, vagal paraganglioma

## Abstract

Paragangliomas are rare tumors of neuroendocrine origin. Within the head and neck, these tumors are slow-growing and locally destructive, with a small malignant potential. Vagal paragangliomas (VPs) originate from paraganglia around the vagus nerve, typically at the level of the skull base. Cranial nerve deficits are common at presentation, with the vagus nerve and hypoglossal nerves being most affected. Similarly, hypoglossal paragangliomas (HPs) originate from around the hypoglossal nerve but are extremely rare and less documented. We describe the case of a patient presenting with an isolated hypoglossal nerve palsy in the setting of a tumor that radiologically represents a VP. A descriptive literature review was conducted to highlight presentation, management, and outcomes related to this pathology.

A 65-year-old male presented to the clinic with tongue fasciculations and several years of dysarthria. Physical examination showed intermittent right tongue fasciculations in addition to ipsilateral hemi-atrophy. A computed tomography scan with contrast revealed an enhancing skull base mass inferior to the right carotid space. Subsequently, magnetic resonance imaging with contrast further delineated its anatomic involvement and site of origin, allowing for the diagnosis of a VP. After further discussion with the patient about his clinical findings, the decision was made to proceed with observation and serial imaging.

Skull base paragangliomas are a rare pathologic entity that may pose a challenging multidisciplinary approach to optimize management strategies. Treatment may vary on a case-by-case basis and is dependent on patient and tumor characteristics.

## Introduction

Paragangliomas are rare neuroendocrine tumors in the head and neck, characterized by slow growth, local destruction, and low malignant potential [1]. These tumors account for 0.6% of all head and neck tumors [2,3]. While typically benign, head and neck paragangliomas, in particular vagal paragangliomas (VPs), exhibit rare malignant potential, and their etiology remains poorly understood.

VPs are derived from the perineurium in the paraganglionic tissue of the vagus nerve and comprise 5% of head and neck paragangliomas [1,4]. Although these tumors may arise from anywhere along the course of the vagus nerve, they typically originate from the inferior nodose ganglion [5]. The clinical presentation of VPs depend on tumor size and location and most commonly present as a painless neck mass. However, cranial nerve deficits are common at presentation with the vagus nerve being most commonly involved [4,5].

In contrast to VPs, hypoglossal paragangliomas (HPs) are exceptionally rare, with fewer than 10 cases reported in the literature [6-11]. However, the proximity of the hypoglossal nerve to the carotid sheath may make a definitive diagnosis based on imaging alone very difficult, as tumors originating from the nerve may mimic VPs or carotid body tumors [11]. Although unilateral tongue weakness and wasting may raise suspicion for hypoglossal nerve origin, its involvement in other skull base paragangliomas makes this diagnosis particularly challenging. Furthermore, a painless neck mass with an indolent growth pattern as a presenting symptom is commonly described in these tumors, imitating VPs [11].

In this report, we describe the case of a patient who presents with an isolated hypoglossal nerve deficit in the setting of a skull base mass radiographically consistent with a VP. The discrepant clinical and radiographic nature of this tumor, to our knowledge, is the first described case of its kind. A comprehensive examination of the current understanding of VPs and HPs is also included.

## Case presentation

A 65-year-old male with a past medical history significant for hypothyroidism, hypertension, dyslipidemia, gastroesophageal reflux disease, and cataracts presented to the otolaryngology clinic for tongue discomfort. Upon further questioning, the patient reported a several-year history of dysarthria, specifically when pronouncing the letter “L.” He also noted occasional tongue biting on the right side. These findings have been stable since onset, and he denied pain, dysphonia, or dysphagia.

**Figure 1:**
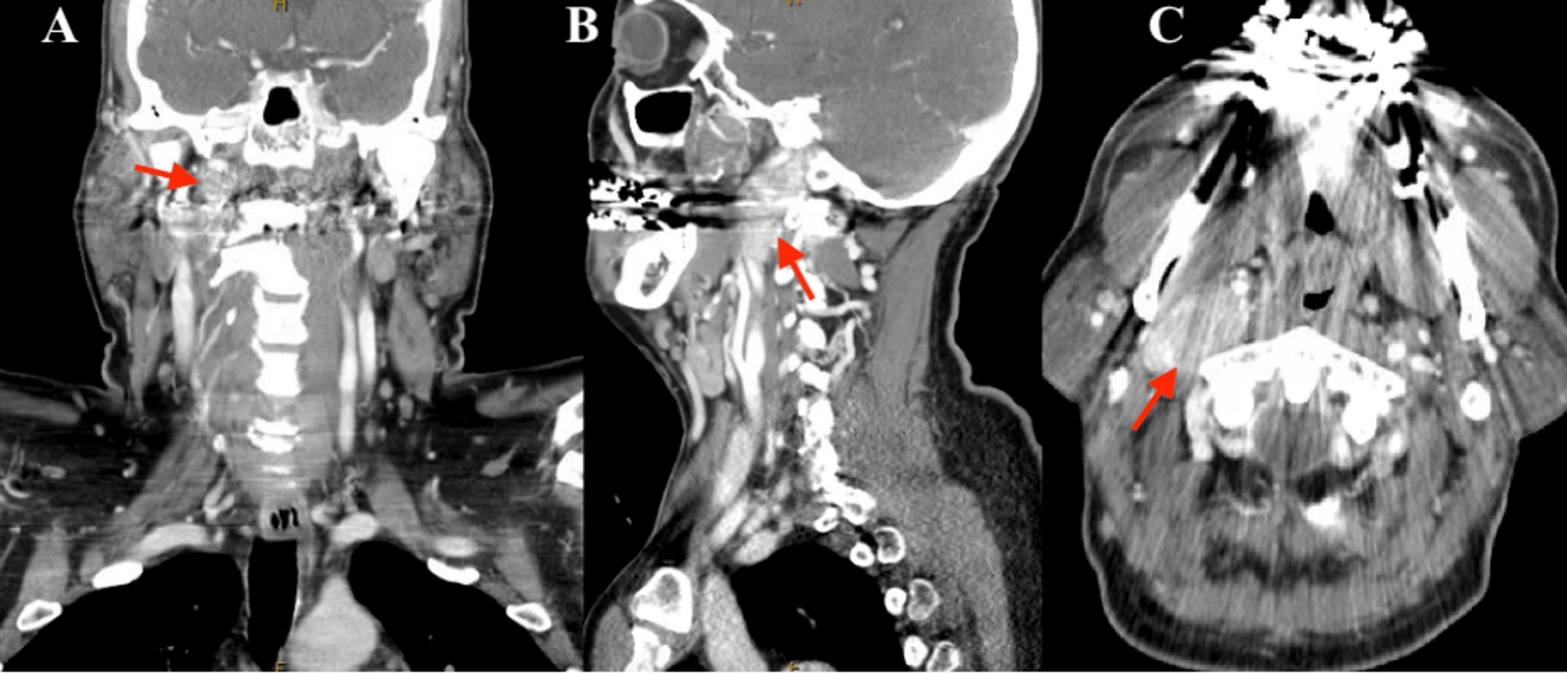
Computed tomography showing an enhancing mass (red arrows) along the right skull base with extension into the jugular foramen and hypoglossal canal. The internal carotid artery is displaced anteromedially. A) Coronal. B) Sagittal. C) Axial.

**Figure 2:**
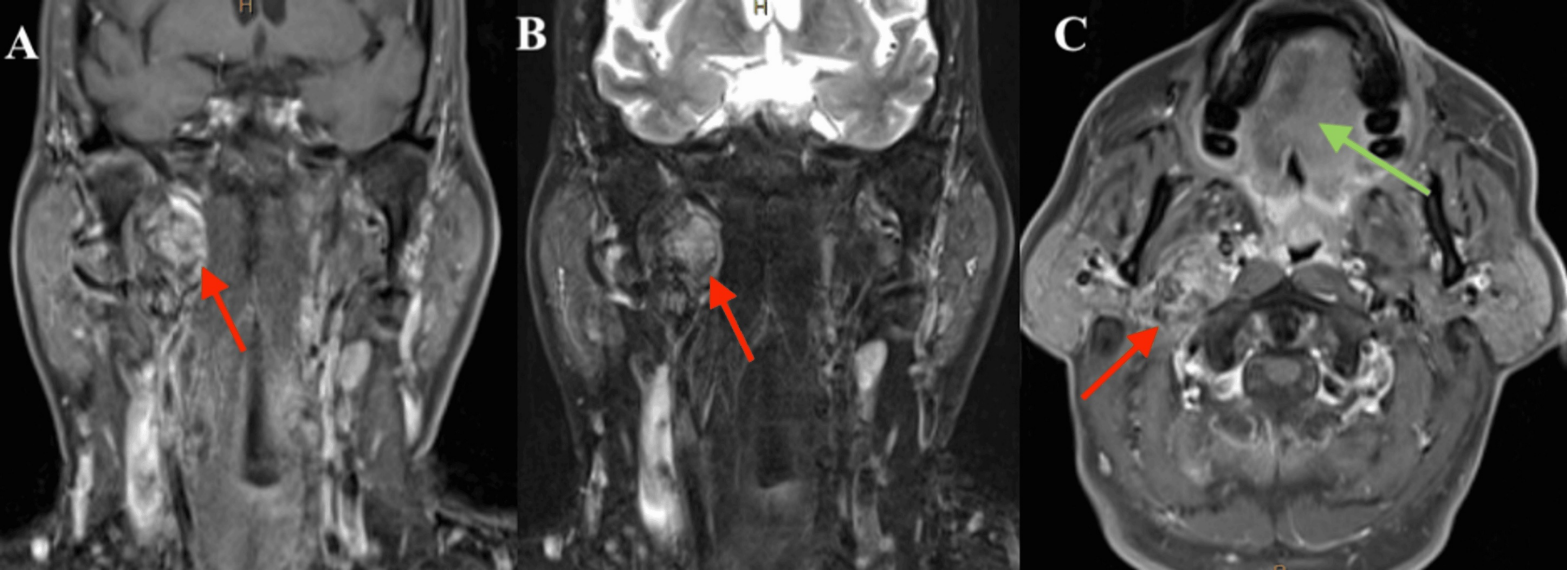
Magnetic resonance imaging depicting a heterogeneously enhancing mass (red arrows) with anterior displacement of the internal carotid artery. The right internal jugular vein flow void is inseparable from the mass. There is notable fatty atrophy and edema involving the intrinsic musculature of the right tongue (green arrow). A) Coronal, T1 sequence post-contrast. B) Coronal, T2 sequence. C) Axial, T1 sequence post-contrast.

Physical examination showed hemi-atrophy of the right tongue as well as ipsilateral fasciculations. CT scan of the neck displayed a heterogeneously enhancing mass centered in the right carotid space (Figure 1). Follow-up MRI of the neck demonstrated an enhancing 4.5 × 2.0 × 5.5 cm mass in the jugular foramen with medial involvement into the hypoglossal canal and inferior extension into the right carotid space. There was noted anterior displacement of the right carotid artery with characteristic intratumoral flow voids (Figure 2) and fatty infiltration of the right tongue with ipsilateral tongue atrophy (Figure 2C).

Due to the unusual incongruity between the clinical and radiographic findings, imaging was reviewed with a neuroradiologist at our institution, and it was determined that the epicenter of the mass was likely stemming off the vagus nerve. Treatment options entailing surgery and education regarding the patient’s clinical situation were discussed in detail. The patient decided not to undergo surgery at the moment to prioritize treatment for other health conditions. The patient is currently being managed with observation and serial imaging, where they will follow up in six months.

## Discussion

Skull base paragangliomas are rare benign tumors that often present in the head and neck region, typically originating near critical structures such as the carotid artery, jugular vein, or cranial nerves. In this report, we present a unique case demonstrating radiologic findings consistent with a VP yet exhibiting clinical signs indicative of an HP. The discrepant nature of these findings underscores the complexity of diagnosing skull base paragangliomas. The following discussion will focus on vagal and HPs, emphasizing the importance of a multidisciplinary approach in accurately identifying and managing these tumors.

Overview and presentation

VPs are rare but well-established entities in the medical literature, with an incidence of approximately one in 100,000 and a slight female predilection. These tumors typically present around the age of 45 years. Up to 50% of cases have a genetic basis, often manifesting with a younger age of onset and multifocality [5,12]. Specifically, genes encoding different subunits of the succinate dehydrogenase (SDH) enzyme complex have been implicated [2]. Among head and neck paragangliomas, VPs are notable for their highest malignant potential [1]. It is worth noting that there is no standardized staging system; instead, malignancy is determined by the presence of regional or distant metastasis, which has been associated with higher mortality rates [1,3,13].

The natural history of VPs remains incompletely understood and depends on tumor size, location, and clinical factors such as involved neurovascular structures. A growing proportion of tumors may be found incidentally, considering the more frequent usage of cross-sectional imaging [2,14]. However, VPs most commonly present as a painless lateral neck mass. Cranial nerve deficits are also common at presentation, with the vagus nerve and hypoglossal nerves experiencing deficits of around 28-37% and 17-21%, respectively [4,5]. Other less commonly involved cranial nerves include the spinal accessory, glossopharyngeal, and facial nerves [4]. Additionally, some patients may experience Horner syndrome based on tumor proximity to the sympathetic nerve pathway [5]. In contrast to paragangliomas present at other sites of the body, head and neck tumors rarely secrete catecholamines, with an estimated incidence of 2-5% [5,15]. Similar to pheochromocytomas, secretory tumors that produce vasoactive substances can cause symptoms of catecholamine excess, typically presenting as hypertension and/or tachycardia. The wide array of clinical symptoms is attributed to the various potential sites of origin along the course of the vagus nerve and the corresponding local involvement.

VPs usually originate from the first 2 cm of the extracranial course of the vagus nerve and most commonly are associated with the inferior vagal or nodose ganglion [16]. They may be limited to the cervical region, be firmly attached to the skull base, or extend intracranially. Some authors believe that the ganglion of origin of the VPs may determine the clinical presentation and growth pattern of the lesion. For instance, lesions arising from the inferior (nodose) ganglion tend to remain limited to the cervical region, whereas tumors from the middle ganglion are cone-shaped and attached to the skull base. Tumors from the superior (jugular) ganglion are usually dumbbell-shaped with intracranial and cervical components.

HPs are exceptionally rare, with fewer than 10 cases reported in the literature [6,9-11]. The radiographic findings seen in our patient were more suggestive of a vagal origin. However, the close proximity of these tumors to the carotid vessels has rendered pre-operative diagnosis exceedingly challenging. Moreover, associated neurological symptoms from brainstem compression have been described and may complicate the clinical picture [7].

To our knowledge, our report described the only case of isolated hypoglossal nerve palsy attributed to a preliminary diagnosis of VP. The presentation of an isolated hypoglossal nerve palsy was acknowledged as being unusual, which prompted a further review of the imaging with the neuroradiologist at our institution. Despite further review, the MRI findings were most suggestive of a VP. The right internal carotid artery was noted to be anteriorly displaced, and the right internal jugular vein flow void was inseparable from the mass. Although there was an extension into the hypoglossal canal, the epicenter appeared to be along the vagus nerve.

Diagnosis and treatment

Initial evaluation begins with a thorough history and physical examination, with special attention paid to cranial nerve function. When suspicion for a head and neck paraganglioma is raised based on clinical signs or symptoms, family history, or incidental findings on imaging, appropriate diagnosis and management become essential.

Imaging characteristics coupled with the appropriate clinical scenario are sufficiently distinctive to permit an accurate diagnosis. A CT scan of the head and neck is an appropriate initial choice as it defines the boundaries of the mass and highlights any local bony destruction within the surrounding architecture [2,13]. A homogenous mass with intense enhancement upon the administration of intravenous contrast is classically seen. MRI is complementary to CT as it allows for enhanced soft tissue detail, which is imperative in the setting of skull base pathology, where critical neurovascular structures exist.

VPs characteristically displace both the internal and external carotids anteriorly, which is in contrast to carotid body tumors where the internal carotid artery is displaced in a posterolateral direction [17]. Evaluation by T1-weighted MRI often reveals paragangliomas with a signal intensity intermediate to surrounding tissues. These lesions frequently demonstrate foci of signal void, attributed to the presence of high-flow vasculature within the tumor. T2-weighted MRI sequences classically show a “salt and pepper" appearance, which manifests as a heterogeneous distribution of signal voids interspersed with regions of focally high signal intensity. It is worth noting that these findings can be observed in other hypervascular tumors and are not specific to paragangliomas [18]. Additionally, in the setting of a newly diagnosed head and neck paraganglioma, some groups advocate for imaging of the thorax, abdomen, and pelvis to exclude metastatic or synchronous paragangliomas using an MRI skull base to pelvis or alternatively a Gallium Ga-68 DOTATATE positron emission tomography (PET) CT [2,19].

Despite the rare incidence of functional paragangliomas within the head and neck, all patients should undergo biochemical testing to avoid precipitating a catecholamine crisis. A catecholamine crisis is a life-threatening emergency where the adrenal gland produces high levels of catecholamines, such as noradrenaline and adrenaline. These high levels can cause induced hypertension that can lead to organ failure and cardiovascular complications. Testing is performed by measurements of urinary and/or plasma fractionated metanephrines and catecholamines. If significant elevations are detected, additional workup may be warranted to evaluate for synchronous paragangliomas located elsewhere in the body.

The only current curative option for head and neck paragangliomas is surgical excision. Observation is also an appropriate treatment route in the suitable patient population. The decision must be made after the consideration of several factors, including the size and location of the tumor, the status of cranial nerve function pre-operatively, and the patient's age and comorbid conditions [12]. Due to the intricate neurovascular structures involved or closely situated to these tumors, their management can present significant challenges. Preserving cranial nerve function through nerve monitoring is paramount in any curative treatment approach. In the case of VPs, surgical intervention inevitably leads to vagus nerve injury, even in the absence of intimate involvement [12]. Intraoperatively diagnosed VPs without pre-operative vagal weakness, especially when initially suspected to be carotid body tumors, may warrant a shift toward nonsurgical management to avoid unnecessary risks of damaging the vagus nerve. The role of radiation therapy for the treatment of VPs has been associated with several serious complications, including skull base osteomyelitis, and currently lacks a strong evidence base [12,20]. By the same principle, the treatment modalities for suspected HPs must be carefully considered in the context of the patient’s clinical condition to avoid unnecessary morbidity. Given the rarity of these tumors and the diagnostic challenges, treatment guidelines have not been established.

## Conclusions

Paragangliomas are rare neuroendocrine tumors that originate from neural crest cells and can occur in various locations within the head and neck. Among these, VPs typically arise from the vagus nerve, presenting frequently as painless neck masses with common cranial nerve deficits, particularly affecting the vagus nerve followed by the hypoglossal nerve. In contrast, HPs are very uncommon and pose significant difficulties because of their close location to the carotid sheath and their resemblance to other skull base tumors. Treatment strategies aim to balance tumor control with the preservation of neurological function. This case report highlights a 65-year-old male with an isolated hypoglossal nerve palsy and a skull base mass radiographically consistent with a VP, marking a unique presentation that underscores the complexities in diagnosing and managing these tumors. A multidisciplinary approach is essential for optimizing treatment, and it must be tailored to individual patients and tumor characteristics, given the absence of established guidelines.
